# Molecular Footprints of *Potato Virus* Y Isolate Infecting Potatoes (*Solanum tuberosum*) in Kenya

**DOI:** 10.1155/2024/2197725

**Published:** 2024-08-06

**Authors:** Maryrose Nyakio, Mariam Were, Clabe Wekesa, Henry Lungayia, Patrick Okoth, Hassan Were

**Affiliations:** ^1^ Department of Biological Sciences School of Natural Sciences Masinde Muliro University of Science & Technology, P.O. Box 190, Kakamega 50100, Kenya; ^2^ Max Planck Institute for Chemical Ecology, Hans-Knöll-Straße 8, Jena 07745, Germany; ^3^ Department of Agriculture and Land Use Management School of Agriculture Veterinary Sciences and Technology Masinde Muliro University of Science and Technology, P.O. Box 190, Kakamega 50100, Kenya

## Abstract

*Potato virus Y* (PVY) is a highly diverse and genetically variable virus with various strains. Differential evolutionary routes have been reported in the genus Potyvirus, caused by natural selection pressure, mutation, and recombination, with their virulence being dependent on different environmental conditions. Despite its significance and economic impact on Solanaceous species, the understanding of PVY's phylogeography in Kenya remains limited and inadequately documented. The study centers on the molecular characterization of a Kenyan PVY isolate, GenBank accession number PP069009. In-depth phylogenetic analysis unveiled a strong evolutionary association between the Kenyan isolate and isolate [JQ924287] from the United States of America, supported by a robust 92% probability. Recombinant analyses exposed a mosaic-like genetic architecture within the Kenyan isolate, indicating multiple gene recombination events. Selection pressure scrutiny identified specific sites under selective pressure, with evidence of positive/diversifying and negative/purifying selection. Population genetics analysis revealed a calculated nucleotide diversity (*π*) of 0.00354881, while analysis of molecular variance (AMOVA) unveiled a structured genetic landscape with an øST value of 0.45224. The extensive haplotype network depicted the possibility of diverse PVY strains occurring across continents. This analysis provides valuable insights into the genetic diversity and distribution of PVY globally, highlighting the importance of understanding evolutionary dynamics for effective management and control strategies of PVY on a global scale.

## 1. Background

Irish potato ranks fourth among the most crucial food crops globally, after maize, rice, and wheat. In Kenya, potato is the second most important food crop after maize. It plays a crucial role in addressing food security and alleviating poverty. Sustainable solutions are required to combat various biotic and abiotic stresses affecting its production, to ensure a stable food supply for the growing population [1, 2]. Among the challenges facing solanaceous crops, Potato Virus Y is a significant and economically impactful threat. The various strains of PVY affect potato production in multiple ways, making it a key focus in the study of potato plant pathogens. Spread by aphids in a nonpersistent manner, PVY poses a threat not only to potatoes (*Solanum tuberosum*) but also to other solanaceous species including tomatoes (*S. lycopersicum*), tobacco (*Nicotiana tabacum*), and nonsolanaceous hosts, such as smooth pigweed (*Amaranthus hybridus*) [3, 4]. PVY genome is a single-stranded positive-sense RNA of approximately 9.7 kb to 11 kb and encodes ten functional proteins, each playing a crucial role in the infection process [5, 6]. The evolution of PVY strains results in new variants with different biological properties that impact potato production globally [4, 7]. Phylogenetic analyses provide significant pieces of evidence information that unveil the degree of evolutionary relationship among genera, species, and phylotypes, the diversity of geographical isolates, and the origin and evolution of different plant viruses [8–10]. Differential evolutionary routes have been reported in the genus *Potyvirus*, caused by natural selection pressure, mutation, and recombination [11].

Potyviruses often cause diverse infections, their virulence being dependent on different environmental conditions [12, 13]. This stems from the parental origin of resistance genes of the host plant and modulation of the proficiency of RNA interference (RNAi) by different environmental conditions. Negative selection is strongly evident in the genes HC-Pro, coat protein (CP), and Nia and Nib genes, while the PIPO gene is the least selected [14]. Mutations and recombination play a significant role in the genetic variation of various PVY strains, potentially leading to more severe symptoms in different crops. Previously, the classification of PVY strains was based on the plant species from which the samples were obtained. This method identified four PVY strains associated with specific hosts: potato, tobacco, tomato, and pepper. However, this classification has become less relevant over time, as some strains have demonstrated the ability to infect multiple host species. In major potato-growing regions worldwide, at least nine distinct strain families of PVY have been identified. This includes nonrecombinant parental strains PVY^O^ (ordinary strain), PVY^C^ (common strain), and PVY^N^ (necrotic strain), which all differ in their biological properties [15, 16]. Several studies have been conducted to highlight the various processes by which different strains induce infections in different hosts [17–19].

In many developing countries where the utilization of certified potato seed systems is limited, there is evidence of seed degeneration and a steady, long-term decrease in yield attributed to viral infections [20–22]. Potato virus Y is incurable under normal field conditions; therefore, known prophylactic measures used in Kenya are focused on preventing or slowing down the viral spread in fields using resistant varieties [23]. The attainment of virus-free propagating material has been realized through molecular procedures. To implement effective control strategies, it is important to obtain molecular-level data on specific viral strains. This is crucial as they may symptomatically reveal epidemiological properties that can aid in accurately diagnosing viral diseases [24].

To effectively control viruses of high economic significance to crop production, efficient detection methods that offer insights into the genomic architecture of the target viruses have to be employed [25]. Numerous strategies have repeatedly endured the test of time as initial screening tools for the presence and absence of viral infections. These include polymerase chain reaction (PCR), reverse transcriptase PCR (RT-PCR), enzyme-linked immunosorbent assay (ELISA), and restriction enzyme analysis. These rely on prior knowledge of viral genomes [26]. Next-generation sequencing (NGS) technologies have proven beneficial for the detection and characterization of viruses in potato plants. They offer insights into viral-host interactions at a molecular level, illuminating both novel and existing pathogenic viruses [27, 28]. In Kenya, next-generation sequencing (NGS) is gaining importance and is being applied in the fields of public health [29–31] and agriculture [32, 33] to mention a few. RNA-Seq is the greatest analytical tool for functional genomics studies, such as differential gene expression, alternative splicing, and variant findings. mRNA sequencing (mRNA-Seq) investigates the transcriptome status, short small RNA sequencing (smRNA-Seq) enables genome-wide profiling and analysis of both novel and known miRNA variants, and whole transcriptome sequencing (WTS) detects coding and multiple forms of noncoding RNA. They are all categorized based on read length [28]. The process of completing cDNA involves three key steps: total RNA isolation, target RNA enrichment, and reverse transcription of RNA into complementary DNA (cDNA). RNA sequencing processes consist of several main components, including RNA stabilization, RNA separation, enrichment, library creation, library controls, barcoding, and RNA sequencing. All the PVY sequences reported to GenBank come from all continents except Antarctica [34] with a vast number of reports signifying a wide range of changing dynamics of viral genome with varying host ranges toward host adaptation.

There is a lack of data on preexisting genomic analysis of *Potato Virus Y* affecting the potato crop in Africa and more so in Kenya. Genome sequencing is crucial because it provides information on the status of different genes along genomes. This method enables the creation of introgression libraries by crossing a cultivated parent with a wild donor, then backcrossing the *F*1 generation with the cultivated parent three to four times, enhancing resilience against PVY. The primary objective of this study was to unveil the molecular phylogeography of Kenyan PVY isolate, providing a foundational analysis of its molecular profile and relative significance.

## 2. Materials and Methods

### 2.1. *Potato Virus Y* Strain Type Sample Collection

A survey conducted in 2018–2019 in Kenya's potato main growing areas, namely, Tigoni, Meru, Molo, Bomet, Uasin Gishu, West Pokot, Trans-Nzoia, and Mount Elgon, provided symptomatic potato leaf samples that were subjected to serological tests. It was done during the long rainy season (March–June rains). Potato fields were selected randomly across several administrative levels: two subcounties per county and two to four wards in each subcounty, depending on the availability of the potato crop on the farm.

**Figure 1:**
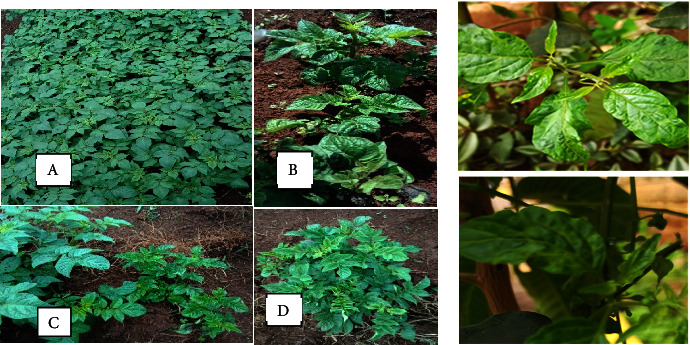
(a) (A) Healthy potato plant in a farm field; (B) potato virus Y-positive potato plant; (C) PVY-positive plant (short with stunted growth) next to a healthy plant; (D) potato virus Y-positive potato plant in the field. (b) Nonsolanaceous host (black nightshade; *Solanum nigrum*) infected with PVY—source: survey data, Kiambu County, Kenya.

Phenotypic symptoms keenly observed for sample collection included stunted growth, chlorotic mosaics on leaves, crinkling of leaves, and deepening venation (Figure 1). The potato leaf samples phenotypically identified were carefully collected and immediately placed in a sterile bag and placed in an ice-filled cool box in the field then transferred to −20° for further laboratory analysis.

#### 2.1.1. Serological Characterization of PVY Samples from Farmer Fields

The collected samples underwent initial testing for the presence of PVY using a double-antibody sandwich ELISA, following the manufacturer's instructions. Adjustments were made as necessary based on troubleshooting efforts. The monoclonal PVY^O/C^, PVY^C^, and PVY^N^ antibodies were employed for differentiating PVY strains. Negative controls in the form of virus-free plant samples of potatoes were included, while a confirmed PVY-infected potato sample obtained from the field served as the positive control.

### 2.2. Molecular Detection of PVY Strains Based on the CP (Coat Protein) and P1 Gene Using Sanger Sequencing

#### 2.2.1. RNA Extraction and Reverse Transcription for Kenyan PVY Strains

RNA extraction was performed on infected plants using the Spectrum Plant Total RNA Kit obtained from Sigma-Aldrich, following the manufacturer's instructions, with slight modifications. Frozen potato leaves were finely ground using a sterile pestle and mortar. The ground tissue was carefully transferred to precooled RNase-free microcentrifuge tubes. Lysis buffer was added to each sample, with a volume of 1.5 ml per 0.25 g of tissue powder. The lysate was homogenized by vortexing to thoroughly disperse the sample, followed by incubation for 3 minutes at room temperature. Subsequently, a volume of 350 *µ*l was transferred to a clean homogenization tube and centrifuged at 13,000 rpm for 5 minutes. To each homogenate, one volume of 70% ethanol was added. The samples were vortexed thoroughly and transferred to a spin cartridge, which was centrifuged at 13,000 rpm for 15 seconds, and subsequently, the flow-through was discarded. The spin cartridge was reinstated into the same collection tube following the previous steps. A repeat of the process was conducted using buffer II, followed by a final spin at 13,000 rpm for 2 minutes to dry the membrane with bound RNA. The purified RNA was then stored at −20°C. To assess the quality of the extracted RNA, one *μ*l of the RNA extract was run on a 1.2% agarose gel. Additionally, the RNA concentration was measured using a NanoDrop (Table 1).

**Figure 2:**
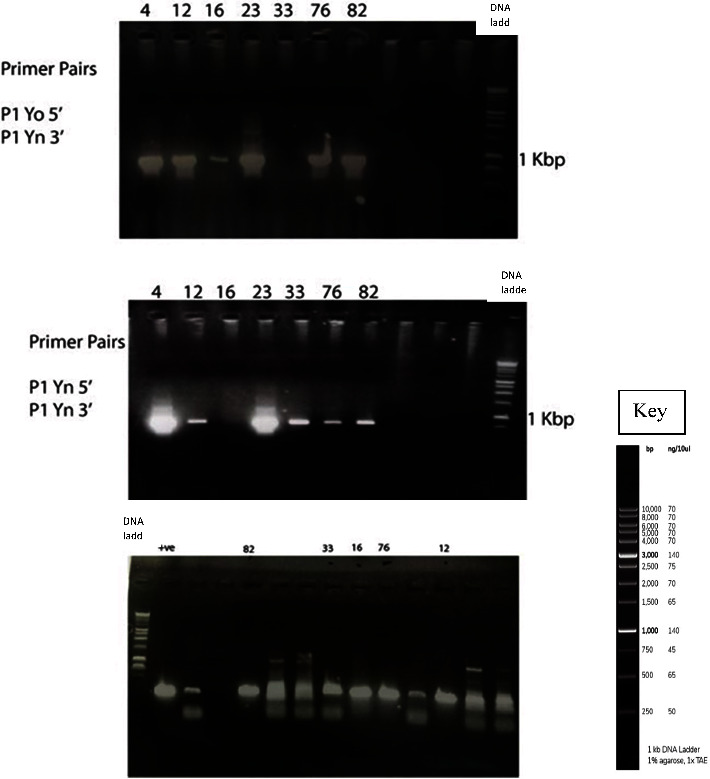
Gel electrophoresis of PCR products of Potato Virus Y (+ve) samples comprising of 1 kbp DNA ladder in the last well, positive control from a verified PVY + ve sample, and different strains of positively identified PVY samples from Kenyan farmer fields [sample ID K4 (Kenya Mpya potato variety), K82 (Sherekea potato variety), K33 (Dutch potato variety), K16 (Dutch potato variety), K76 (Dutch potato variety), and K12 (a screen house potato variety)].

The reverse transcription (RT) was synthesized in a 25 *µ*l reaction mix utilizing M-MLV reverse transcriptase from Thermo Scientific. The reaction mix comprised of 1 *µ*l cDNA template, 0.5 *µ*l of forward and reverse primers (Table 2) that were designed, 0.5 *µ*l of 10 mM dNTP, 5 *µ*l of 10X Taq polymerase buffer, 0.25 *µ*l of Taq DNA polymerase (5 U/l), 1.5 *µ*l of MgCl2, and 15.75 *µ*l ddH_2_O composed the PCR Master mix. The PCR cycle was configured to start with a 2-minute denaturation step at 95°C, followed by 30 cycles consisting of denaturation at 95°C for 30 seconds, annealing at 58°C for 1 minute, and extension at 72°C for 2 minutes. Finally, there was a single extension step of 5 minutes at 72°C. The amplification results were separated on a 1.2% agarose gel in 1X TAE buffer (as shown in Figure 2), stained with 0.75 *μ*l of GelRed (from Biotium, USA).

#### 2.2.2. Cloning and Sequencing of the PCR Fragments for PVY Strain Identification

High-fidelity polymerases cloned for sequencing were used to create multiplex-PCR fragments. The Thermo Scientific GeneJET PCR Purification Kit was utilized to purify PCR products, and the cDNA content was quantified using a NanoDrop. For further validation of purity and concentration, 5 *µ*l of purified cDNA was subjected to electrophoresis on a 1.2% agarose gel. Following ligation, the purified PCR product was inserted into the pGEM-T cloning vector using the CloneJET PCR Cloning Kit from Thermo Scientific. Subsequently, the resulting construct was transformed into competent cells of *Escherichia coli* strain DH5 obtained from Life Technologies, following the manufacturer's instructions.

Plasmid DNA was then extracted from overnight cultures of selected *E. coli* colonies using the Thermo Scientific GeneJET Plasmid Miniprep Kit. The extracted plasmid DNA was digested with FastDigest BgIII enzyme from Thermo Scientific, and the digests were examined using agarose gel electrophoresis. Colonies exhibiting the expected insert sizes were selected for sequencing using the Sanger sequencing technique. Each sample and PCR result had one to three clones sequenced.

### 2.3. *Potato virus* Y Sample Collection for Whole-Genome Sequencing Using RNA-Seq

Three symptomatic potato leaf samples were obtained from the same plant at Tigoni Potato Research Farm, managed by the Kenya Agricultural and Livestock Research Organization (KALRO). The farm is situated at a latitude of 1°080′S and a longitude of 36°400′E, with an altitude of approximately 2100 meters above sea level.

Upon collection, the samples were promptly placed in properly labeled Falcon tubes containing RNAlater® (RNA stabilizing solution). To maintain their integrity, the samples were stored in a cool box while still in the field. Later, they were transported to the BecA-ILRI Hub (Biosciences Eastern and Central Africa-International Livestock Research Institute) for further experimentation under controlled conditions at 4°C.

#### 2.3.1. Sample Preparation

Total RNA extraction was performed using a RNeasy Plant Mini Kit, adhering to the manufacturer's protocol with slight modifications.

#### 2.3.2. Library Preparation

Library preparation followed the Illumina TruSeq low sample preparation protocol with minor modifications. Fifteen microliters of Elute Prime Fragment mix, containing random hexamers for RT priming and first-strand cDNA synthesis buffer, were added to sterile PCR tubes containing ten microliters of 500 ng RNA obtained from the previous step. The mixture was gently pipetted, mixed thoroughly, and then placed in a preprogrammed thermocycler at 94°C for eight minutes. Subsequently, the thermocycler was held at 4°C to allow for elution, fragmentation, and priming of the RNA. Following this step, the thermocycler immediately progressed to synthesize first-strand cDNA.

50 *µ*l of the SuperScriptII was added to the entire stock (one-microliter SuperScriptII for each 9 *µ*l of the First-Strand Master Mix) to the First-Strand Master Mix tube, mixed gently but thoroughly, and centrifuged at maximum speed for 30 seconds. 8 *µ*l of the First-Strand Mix containing SuperScriptII was added then entire volume to 17 *µ*l of the primed mix and gently pipetted up and down six times, spun down, and placed in a preprogrammed thermo cycler at 25°C for ten minutes, 42°C for fifty minutes, and 70°C for fifteen minutes, and held at 4°C with preheated lid option set to 100°C. This immediately progressed to synthesize the second strand. The thawed Second-Strand Master Mix was centrifuged at 600xg for five seconds. 25 *µ*l was added to the products of the first-strand cDNA synthesis, the entire volume pipetted, mixed thoroughly, spun down, and then incubated in a preheated thermo cycler for one hour.

To purify the double-stranded (ds) cDNA, 90 *µ*l of well-mixed AMPure XP beads was added to the PCR tubes containing 50 *µ*l of ds-cDNA at room temperature. The mixture was thoroughly mixed and then transferred to sterile 1.5 ml labeled microcentrifuge tubes. Subsequently, the tubes were incubated at room temperature for fifteen minutes. After the incubation period, the microcentrifuge tubes and their contents were placed on a magnetic stand at room temperature for five minutes. This allowed all the beads to bind to the side of the tubes due to the magnetic field. Following magnetic separation, the supernatant containing impurities was carefully removed from each well and discarded, while the microcentrifuge tubes remained on the magnetic stand. Without disturbing the pellet, a hundred microliters of freshly prepared 80% EtOH were added and incubated at room temperature for 30 seconds. The 80% EtOH washes were repeated twice.

After the supernatant containing impurities was removed and discarded, the microcentrifuge tubes containing the pellet were allowed to stand at room temperature to dry briefly. A thawed and centrifuged resuspension buffer (RSB) was then added to each tube after its removal from the magnetic stand. The entire volume was gently pipetted ten times (up and down) to ensure thorough resuspension of the pellet. Subsequently, the tubes were incubated at room temperature for two minutes to allow for proper rehydration of the pellet. The microcentrifuge tube was placed in a magnetic stand for five minutes with the cap open. Fifty microliters of supernatant (ds-cDNA) was removed from each microcentrifuge tube and transferred to clean empty PCR tubes. Ten microliters of resuspension buffer was added to each tube, followed by 40 microliters of End Repair Mix. The tubes were then placed in a preheated thermal cycler at 30°C for thirty minutes.

Additionally, 100 microliters of End Repair Mix was transferred to a 1.5 ml microcentrifuge tube. To this, 160 microliters of well-vortexed AMPure XP beads was added, and the entire volume was gently pipetted and thoroughly mixed. Subsequently, the mixture was incubated at room temperature for fifteen minutes. After the incubation step, the tube was placed on a magnetic stand at room temperature for five minutes, allowing the liquid to become clear. Subsequently, 127.5 *µ*l of the supernatant was carefully removed and discarded. While the tubes remained on the magnetic stand, 200 *µ*l of freshly prepared 80% EtOH was added over the pellet carefully, without disturbing it. The tubes were then incubated at room temperature for 30 seconds. The supernatant was carefully removed and discarded without disturbing the pellet. The wash step was repeated once more. Carefully, the tubes were air-dried at room temperature for fifteen minutes while still on the magnetic stand. Next, 20 *µ*l of resuspension buffer (RSB) was added into the air-dried tubes and gently pipetted up and down to mix thoroughly. The entire volume was mixed thoroughly and incubated at room temperature for two minutes. The tubes were placed back on the magnetic stands at room temperature until the liquid became clear. Twenty microliters of supernatant was transferred to a new sterile PCR tube, followed by the addition of 2.5 *µ*l of RSB and 12.5 *µ*l of thawed A-tailing mix. The entire volume was gently pipetted up and down ten times to ensure thorough mixing. The mixture was then spun down and placed on a preprogrammed thermocycler at 37°C for thirty minutes, followed by 70°C for five minutes, and a hold temperature of 40°C with a preheat lid option. The libraries were quantified using Qubit High Sensitivity Kit and quality checked on 2% agarose gel and Agilent Technologies tape station with sensitivity D 1000 screen tape to select libraries. The libraries were diluted to a suitable concentration in resuspension buffer, with dilutions ranging between 4 nM to 10 nM. Two pools were generated from the 4 nM libraries. A sample sheet was generated for each pool in the Illumina experiment manager. These were further diluted to a final concentration of seven pM and loaded to an Illumina TruSeq system for sequencing.

### 2.4. Recombination Analysis

To investigate the role of recombination in PVY evolution, two different approaches were employed for recombination analyses. These approaches were utilized to identify potential recombination events in PVY sequences concerning the Kenyan isolate PP069009. We identified potential recombinant and parental sequences using seven different algorithms, including RDP, GENECONV, BOOTSCAN, MAXCHI, CHIMAERA, SISCAN, and 3SEQ. Default settings were used throughout. RDP (recombination detection program) applies several recombination detection and analysis methods expanding an array of recombination event detection, recombination breakpoint demarcation, and recombinant sequence identification methods, all applied in unison, to yield detailed descriptions of how recombination may have impacted the evolution of any given set of aligned nucleotide sequences [35]. GENECONV examines an alignment of multiple sequences pairwise and scans for abnormally long regions of high identity between the focal pair, contingent on the variable site pattern in other sequences. GENECONV employs codon polymorphisms rather than site polymorphisms, as silent sites within the same codon position are expected to be correlated [36]. The BOOTSCAN algorithm investigates the depths of recombinant regions within the alignment, as determined by crossover points identified through boot scanning. These regions are subsequently analyzed separately through phylogenetic analysis [37]. The MAXCHI function implements the maximum chi-square (MaxChi) method, which is utilized for detecting recombination breakpoints [38]. CHIMAERA is an algorithm designed for the accurate detection and estimation of subclone frequencies. It utilizes whole-exome sequencing and whole-genome sequence data obtained from multiarea biopsies, enabling subclones to be sorted in an evolutionary tree structure [39]. SISCAN assesses phylogenetic and compositional signals in various patterns of identity that occur between four nucleotide sequences [40]. 3SEQ is a command-line program designed to read in a nucleotide sequence file, typically in Philip or aligned FASTA format. It tests all sequence triplets in the file to detect a mosaic recombination signal, indicating that one of the three sequences (the child) is a recombinant of the other two (the parents). The statistical test employed is a nonparametric test for mosaicism, and *p* values are precomputed once using the 3SEQ executable [41].

### 2.5. Phylogenetic Analyses of PVY Strains of Kenya

**Figure 3:**
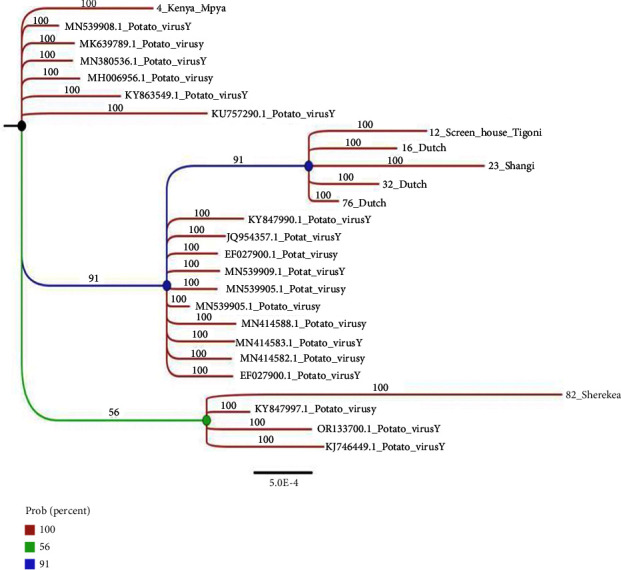
Phylogenetic tree of different PVY variants identified in mixed infection reconstructed from partial coat protein sequences and selected reference genomes using MrBayes program, visualized with Fig Tree program.

**Figure 4:**
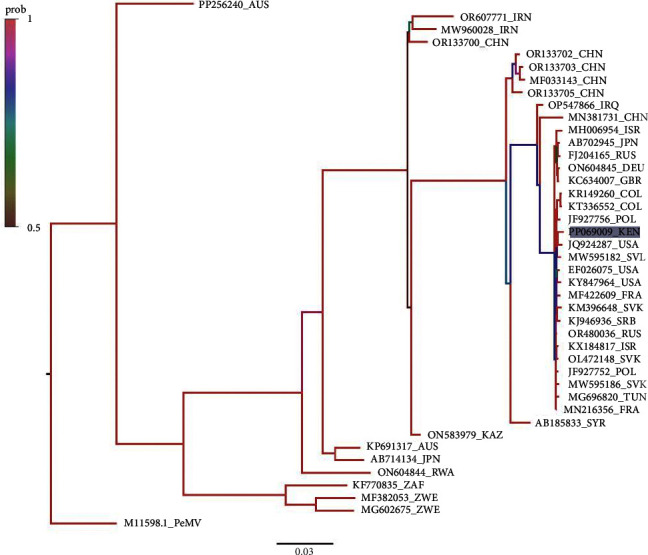
Genetic relatedness and evolutionary connections of the PVY_Kenya isolate within the broader context of global PVY diversity, providing crucial insights into its phylogenetic positioning and potential historical transmission patterns across continents using the MrBayes program, visualized with the Fig Tree program.

Phylogenetic analysis was conducted using partial genome sequences that included complete P1 and CP (coat protein) nucleotide sequences enough to distinguish between the PVY strain types collected from farmer potato fields in Kenya. These isolates are represented by the accession numbers OR571473, OR571477, OR571474, OR571476, OR571478, OR571479, and OR571475 (refer to Table 3). Additionally, the Kenyan PVY whole-genome isolate with accession number PP069009 was included in a further analysis with a selection of sequences representing diverse countries and continents, sourced from the first 100 sequences obtained from the NCBI search. These sequences were aligned using MAFFT software [42], and the phylogenetic tree (Figures 3 and 4) was constructed using MrBayes [43]. Subsequently, the phylogenetic tree was visualized using the Figtree program.

### 2.6. Selection Pressure of the Kenyan PVY Whole-Genome Isolate

#### 2.6.1. Branch Selection

The notable focus on identifying instances of robust selection has led to a methodological gap concerning biologically intriguing cases where the absence or reduction in the effectiveness of natural selection is significant. In this study, the examination of selection pressures within the Potato Virus Y (PVY) phylogeny necessitated a thorough analysis utilizing the aBSREL [44] and RELAX [45] methods. This analysis aimed to discern sporadic diversifying and relaxed selection, respectively, with particular emphasis on the PVY_Kenya branches compared to other phylogenetic groups. Employing a stringent significance threshold (*p* ≤ 0.05) and correcting for multiple testing using the likelihood ratio test, the study expected to identify distinct selective pressures acting specifically within PVY_Kenya branches.

#### 2.6.2. Site Selection Tests

The advent of computationally tractable codon-substitution models [46] has sparked increased scientific interest in natural selection acting on protein-coding genes. Positive selection is inferred when the estimated ratio (*ω*) of nonsynonymous (*β*) to synonymous (*α*) substitution rates significantly exceeds one [47, 48]. This phenomenon is more readily observed in smaller alignments.

A mixed-effects model of evolution (MEME) belongs to the broader class of branch-site random-effects phylogenetic methods [49], and it was the most preferred in this study for it allows the distribution to vary from site to site (the fixed effect) and also from branch to branch at a site (*ω*), reliably capturing the molecular footprints of both episodic and pervasive positive selection [50]. It also matched the performance of traditional site methods when natural selection is pervasive, reliably identifying episodes of diversifying evolution. In the investigation of pervasive positive/diversifying and negative/purifying selection within the Potato Virus Y (PVY) genome using the FUBAR [51], compelling evidence emerged regarding the distribution and nature of selective pressures across multiple sites.

## 3. Results

### 3.1. PVY Strains' Sample Processing

For the analysis of P1 and CP sequences, RNA extractions were done, and their concentrations were estimated using a NanoDrop spectrophotometer. Most sample extractions yielded satisfactory concentration readings (Table 1). These extracted RNA samples were then cloned into pGEM-T vectors to obtain complete P1 sequence data. Before sequencing, PCR products were visualized through gel electrophoresis (Figure 2).

PVY accessions listed in [52] were utilized for the initial identification of PVY strains, with the caveat that additional sequence data are necessary for definitive assignment.

### 3.2. Phylogenetic Analysis of Different Potato Virus Y Strains Sampled from the Farmer Fields

The core sources accountable for evolutionary changes within positive-strand RNA viruses are recombination, reassortment, and accumulation of mutations. Different clades of the tree comprised different strains of PVY from different parts of the world. Distinctively, four monophyletic groups were observed from the original speciation event. The Kenyan isolate K4 (PVY^n:o^ recombinant type) shared the oldest common ancestor with samples from MN539908.1, MN380536.1, and KY863549.1 (collectively from Egypt); MK639789.1 (Kazakhstan); and KU757290 (Brazil), illustrating maximum support by 100% probability, suggesting a possible close revolutionary relationship in comparison with the rest of the isolates included in this analysis (Figure 3). The second clade of isolate K12, K16 K23, K33, and k76 (All Kenyan PVY strain isolates with partial sequences) shared the most recent common ancestor and are portrayed to have possibly undergone the most recent speciation event lately in comparison with the rest included in the study, while still displaying a maximum support probability of 91%. This also portrayed a possible evolutionary relationship with the samples KY847990.1, JQ954357.1, and EF027900.1 (Britain); MN539909.1 and MN539905.1 (Egypt); and several isolates from France (MN414588.1, MN414583.1, and MN414582.1) and EF027900.1 (United Kingdom) where they shared the second common ancestor from the root node, still supported with a good probability of 91%. However, there is a possibility of a difference in time of evolution indicated by the lengths of the branch extensions from the oldest common ancestral node. This clade comprised majorly of PVY strain samples belonging to the ^N^ or ^O-N^ recombinant type. The last clade comprising KJ746449.1 (Poland), OR133700.1 (China), KY847997.1, and Sherekea (Kenyan PVY recombinant strain) shared the most recent common ancestor and distinctively had a lower similarity index supported by a 56% probability from the rest of the clades, a possible indication of an evolutionary relationship that was divergent in comparison with the other clades revealed in the study.

We found the recombinant PVY strain type PVY^N^ and PVY^N:O^ (Table 3) to be prevalent in the area of study sampled.

### 3.3. Phylogenetic Analysis of the Whole Genome of Potato virus Y Kenyan Isolate

To delineate the evolutionary relationships and contextualize the genetic divergence of the *Potato virus Y* (PVY) sample isolated from Kenya, a comprehensive phylogenetic analysis was undertaken with PVY accessions from Europe, Asia, Australia, and Africa, in efforts to further demonstrate the genealogy of the virus on a larger world scale level using MrBayes program (Figure 4), with a pepper mottle virus, accession number M11598 as the root. PP256240 from Australia shared the oldest common ancestor at the second node. This is true for the rest of the clades in this phylogenetic analysis, along with other included accessions.

In the analysis involving accessions from Africa, MF382053 and MF602675 from Zimbabwe were observed to be more closely related to PVY samples from South Africa (KF770835) and Rwanda (ON604844). This was determined based on them sharing the most recent common ancestor. Conversely, the Kenyan isolate PP069009 used in this study showed the highest probability of undergoing the most recent speciation event.

PVY_Kenya isolate (GenBank accession number PP069009) demonstrated a close evolutionary relationship with an isolate from the United States of America (JQ924287) with a maximum support at 92% probability. Regarding isolates present in Africa, the phylogenetic analysis revealed a close association between the most recently shared common ancestry with a representative from Tunisia (MG69820) in comparison to isolates from the rest of Africa.

### 3.4. Recombination Analysis

The recombinant analysis conducted for the phylogeny of *Potato virus Y* (PVY) revealed a mosaic-like genetic structure within the PVY Kenya isolate. Employing the GENECONV method, multiple instances of gene recombination events were identified across the genome, denoted by the specific gene segments (Start-End) involved. Notably, a significant number of these events, spanning various regions, yielded a *p* value of zero, indicating an exceptionally high statistical significance. Furthermore, while most events exhibited a *p* value of zero, a few instances displayed slightly higher yet still relatively low *p* values, suggesting potential recombination events with slightly reduced statistical significance. The analysis highlights the complex nature of genetic exchanges within the PVY_Kenya genome, underscoring substantial genetic diversity arising from recombination events with multiple parental sequences. Notably, specific genomic regions exhibited varied strengths of evidence for recombination, further emphasizing the intricate mosaic pattern characterizing the genetic makeup of the PVY_Kenya isolate.

### 3.5. Selection Pressure

#### 3.5.1. Branch Selection

Selective pressures are the factors that influence the survival and reproductive success of an organism in its environment and are crucial in shaping a population's genetic makeup through natural selection. However, contrary to expectations, neither aBSREL nor RELAX detected statistically significant evidence of episodic diversifying selection or relaxed selection within the PVY_Kenya branches or among the other groups in the phylogeny. This underscores the complexity of selective forces shaping PVY evolution and highlights the need for further detailed investigations into the dynamics of selection within viral populations.

#### 3.5.2. Site Selection Test

**Figure 5:**
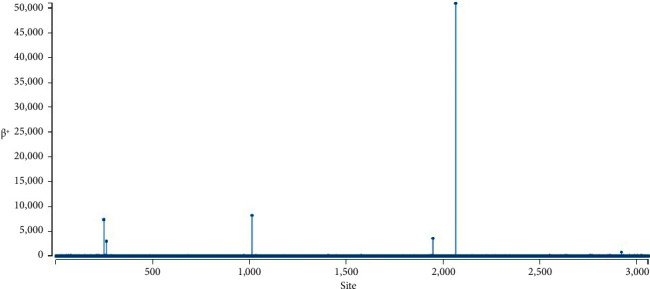
Demonstration of episodic positive/diversifying selection within the *Potato virus Y* (PVY) genome exhibiting selective pressure at 2066 position, at a (*p* < 0.05) using the multiple expectation maximizations for Motif Elicitation (MEME) program.

In the exploration of episodic positive/diversifying selection within the *Potato virus Y* genome, the MEME [50] method was employed, revealing evidence of selective pressure at a specific site 2066, with statistical significance set at a *p* value threshold of 0.05, as illustrated in Figure 5. Specifically, MEME identified a key parameter, *β*+, as pivotal in distinguishing between null and alternative models. In the null model, both *β*+ and *β*− are constrained, whereas in the alternative model, *β*+ remains unrestricted. Positive selection at individual sites is inferred when the *β*+ parameter exceeds *α*+ and is further validated as significant through the likelihood ratio test. This signifies that site 2066 exhibited characteristics indicative of positive selection, highlighting its potential importance in the adaptive evolutionary process of PVY.

**Figure 6:**
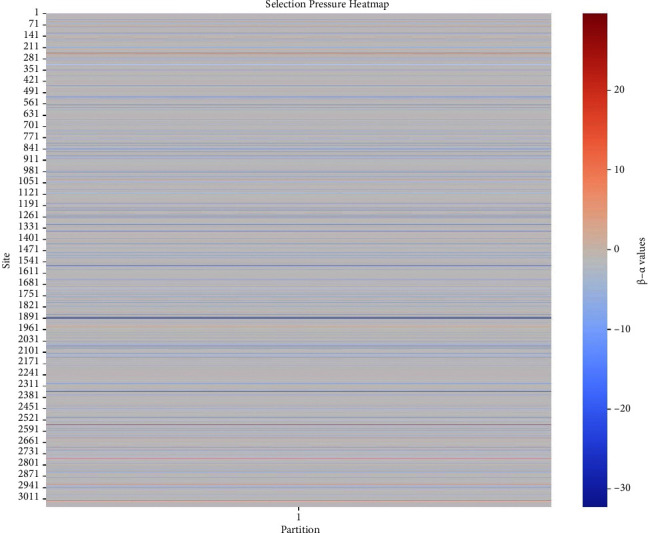
Pervasive positive/diversifying and negative/purifying selection within the *Potato virus Y* (PVY) genome detected at 267 sites, each with a posterior probability (Prob [*α* > *β*]) exceeding 0.9 using Fast, Unconstrained Bayesian AppRoximation for Inferring Selection (FUBAR) model.

The analysis was conducted under the general time-reversible (GTR) model, complemented by the specific model fitting (AICc = 31645.59, log *L* = −15721.77). FUBAR detected pervasive positive/diversifying selection at five distinct sites and pervasive negative/purifying selection at 267 sites, each with a posterior probability (Prob [*α* > *β*]) exceeding 0.9. Notably, positive selection sites clustered predominantly toward the end of the alignment (Figure 6), while purifying selection was concentrated at the beginning.

This result unveiled the complex interplay of selective forces shaping the genetic landscape of this viral population. The notable clustering of sites under positive selection toward the terminal regions of the alignment and the concentration of sites experiencing purifying selection at the beginning suggests a spatially distinct pattern of evolutionary pressures along the PVY genome. This spatial distribution might imply a differential functional significance across the viral genome, where regions toward the end potentially experience more adaptive changes, likely associated with factors, such as host interactions, immune evasion, or adaptation to specific environmental conditions. Conversely, the regions exhibiting purifying selection at the start might encompass critical genetic elements vital for the virus's replication, structural integrity, or conserved functional domains essential for its life cycle. Such divergent selective pressures across the genome could signify a delicate balance between adaptive evolution, where beneficial mutations confer advantages, and the preservation of essential genomic elements to maintain viral fitness. These findings underscore the dynamic nature of PVY evolution, emphasizing the pivotal role of selective pressures in shaping the genetic diversity and adaptability of this virus, thereby providing valuable insights for understanding its evolutionary strategies and potential implications for disease management and control.

### 3.6. Population Genetics Analysis of Potato virus Y (PVY) Phylogeny

An alignment of *Potato virus Y* (PVY) sequences was generated using the Muscle program v5 [53] and subsequently analyzed for haplotype with DnaSP v6 [54]. The program allows the thorough characterization of the levels and designs of DNA sequence disparity at diverse time scales, using polymorphic variants (intraspecific data), divergence data (interspecific or interpopulation data), or a combination of both [54]. It also allows for the analysis of different recreations under a wide array of demographic circumstances. Analyzing PVY samples from distinct continents—America, Africa, Asia, and Europe—revealed a complex genetic landscape reflective of the virus's worldwide distribution and diversity. The calculated nucleotide diversity (*π*) of 0.00354881 underscored a relatively low overall genetic diversity within the PVY population, despite its multicontinental origins. The identification of 290 segregating sites, with 53 being parsimony-informative, highlighted substantial variations across the viral genome, showcasing the diverse genetic makeup of PVY strains originating from various countries within each continent. Tajima's D statistic further illuminated the population dynamics, indicating a value of −2.26205, suggesting potential departures from neutral evolution (Table 4).

This finding may imply various evolutionary pressures acting upon PVY populations across continents, potentially leading to an excess of low-frequency polymorphisms or recent population growth within specific geographical regions. Subsequent analysis using the analysis of molecular variance (AMOVA) unveiled a structured genetic landscape within the PVY population across continents. The ø_ST_ value of 0.45224, indicative of genetic differentiation among continents, portrayed a moderate yet discernible level of differentiation between the PVY strains originating from America, Africa, Asia, and Europe. The fixation indices (Phi_ST, Phi_SC, and Phi_CT) provided a comprehensive understanding of genetic variance across different hierarchical levels. Phi_ST (0.45224) demonstrated substantial genetic differentiation among continents, while Phi_SC (0.39131) indicated genetic variation among populations within continents, albeit to a lesser extent. However, Phi_CT (0.10010) did not achieve significance (*p*=0.161), implying limited variation among continents relative to the total genetic diversity observed. Significance testing of these indices reaffirmed the observed genetic structure, with both Phi_ST (*p* < 0.001) and Phi_SC (*p*=0.006) showing significant values, indicating tangible genetic structure among continents and populations. These findings underscore a structured genetic landscape within the PVY population across continents, characterized by moderate differentiation among continents, low nucleotide diversity, and potential departures from neutrality. This multicontinental analysis emphasizes the need for further investigations into the specific evolutionary dynamics and environmental pressures shaping PVY populations within distinct geographical regions, essential for developing tailored management and control strategies on a global scale.

#### 3.6.1. Haplotype Networks

**Figure 7:**
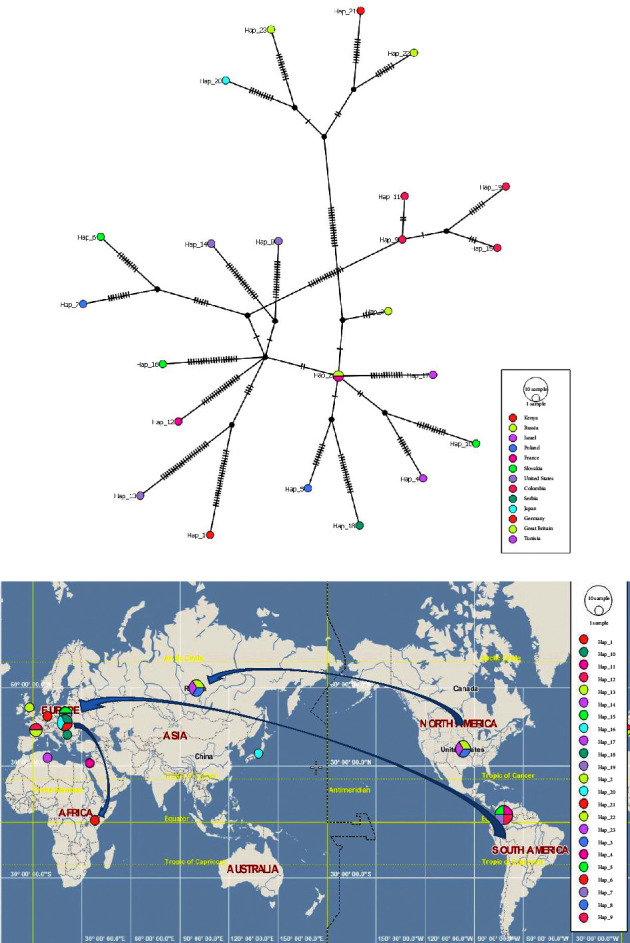
(a) Haplotype networks of selected *Potato virus Y* genome sequences with country color codes denoting their geographical origins around the world. Hap_1 represents the PVY of Kenya revealing a close association with Hap_13 from the United States of America. (b) Haplotype maps providing insights into the genetic diversity and distribution of PVY strains across different continents.

Haplotype networks are used in the analysis of population genetic data to visualize genealogical relationships at the intraspecific level, as well as to make inferences about the biogeogphraphy and history of different populations of organisms [55] and thus defining them [56]. PopArt embraces the least spanning, median-joining, and TCS network methods, as well as AMOVA [57] and Tajima's D statistic [58]. With the primary function of POPART being to infer and visualize genetic relationships among intraspecific sequences, an extensive haplotype list was generated from the DnaSP v6 and further analyzed with PopArt 1.7.2 [55] program to generate haplotype networks and maps. The resulting list depicted a diverse spectrum of haplotypes, each characterized by a unique combination of sequences identified by accession numbers and country codes, denoting their geographical origins. The frequencies associated with these haplotypes provided valuable insights into the genetic diversity and distribution of PVY strains across different continents—America, Africa, Asia, and Europe. The diverse array of haplotypes signifies the potential evolutionary divergence or recombination events within the PVY population. This information served as crucial input for constructing phylogenetic or haplotype networks (Figure 7(a)) and maps (Figure 7(b)) using the PopArt program, enabling visual representations that illustrate the genetic relationships among PVY types from various geographic locations. Overall, 23 haplotypes were identified based on the PVY sequences drawn from different parts of the world (Figure 7(a)). Haplotype 1 is comprised of the Kenyan whole-genome sequence PP069009. PVY sequences from Russia and France were found in haplotype 2. Haplotypes 9, 11, 15, and 19 were from Colombia. Haplotypes 5 and 7 were traced back to Poland. Haplotypes 6, 10, and 16 were from Slovakia. These networks and maps are expected to offer comprehensive insights into the evolutionary history, migration patterns, and sources of genetic diversity within the global PVY population, contributing significantly to our understanding of the virus's dynamics and adaptation across diverse environments.

## 4. Discussion

Pathogen adaptability is inevitable for better survival to the advanced resistance of hosts through mutations, recombination, or gene flow [59]. Loss of genetic purity, gene erosion, and accumulation of pathogens in the seed potato have contributed to fluctuations observed in the global scales of potato production [20, 60]. Accurate extrapolation of the evolutionary course of pathogens, evaluating population dynamics, and thoughtful considerations of population genetic structure work together toward achieving plant populations that are resistant or tolerable to different biotic and abiotic factors without causing crop loss, which has repercussions to the world population.

The phylogenetic analysis provides crucial insights into the evolutionary relationships among genera, species, and phylotypes, revealing the diversity of geographical isolates and the origin and evolution of plant viruses [8–10]. The Incas, the first South American farmers, initiated potato farming, and the domestication of arable Irish potatoes originated from wild potato species crossings in the Andean region [61]. The introduction of arable Irish potatoes to North America and Asia from South America dates back to the late 15th and 16th centuries, facilitated by seed exchange with Colombia, eventually spreading to other nations [62, 63].

Reports from the NCBI GenBank database reflect the changing dynamics of viral genomes with varying host ranges and host adaptation. Despite the efficacy of seed certification methods in reducing viral infections, *Potato virus Y* (PVY) remains a significant issue in potato seed production, impacting yields and tuber quality. The spontaneous evolution of new PVY strains poses challenges to certification and farm management measures, raising concerns in potato production.

PVY sequences in GenBank span all continents except Antarctica [34], yet information on the molecular footprints of PVY in Africa is scarce. This study conducted comprehensive phylogenetic analyses using the NCBI Nucleotide Blast and a curated sequence dataset, including the PVY_Kenya genome and sequences from countries in the continents of Asia, Africa, Europe, North America, and Oceania. Phylogenetic analysis revealed a shared common last ancestor node between the PVY_Kenya isolate (GenBank accession number PP069009) and isolates from the United States of America (JQ924287), with maximal support at 100% probability. This positioning suggests a close evolutionary relationship between PVY_Kenya and its United States of America counterpart, potentially tracing back to a common ancestral lineage rooted in Slovakia. Our findings indicate a migration trail from South America to Europe, possibly introducing PVY to Europe from South America, with subsequent spread to Africa (Figures 7(a) and 7(b)).

The current study sought to do a comprehensive phylogenetic analysis of *Potato virus Y*, utilizing the NCBI Nucleotide Blast of curated sequence dataset comprising the partial sequences of the Kenyan PVY, that saw the amplification of the P1 (full sequence) and coat protein region of isolates from the farmer fields and the whole genome of PVY_Kenya. Seven potato plants of different cultivars collected across the major potato-producing counties that tested positive for PVY exhibiting foliar mosaic, crinkling, and mottle were collected in seed potato production and were sequenced to reveal the existence of difference in strains with the Kenyan PVY strains. Seven different algorithms were used in this study, and we identified potential recombinant and parental sequences (Figure 3). There has been an evolutionary shift detected over the years resulting from the genome recombination of two of the traditionally known strains (PVY^O^ and PVY^N^) to new recombinant strains, predominantly PVY^NTN^ and PVY^N−Wi/N:O^ [16, 64, 65] in different parts of the world.

The spatial population structure of plant pathogens differs from one area to another due to different biotic factors like host genetics and abiotic factors, for example, the environment. Extreme resistance (ER) in potato is expressed by Rysto and it averts viral replication without causing cell death because it associates substantially with PVY coat protein. However, when the *Potato virus Y* coat protein is overexpressed, it can prompt macroscopic hypersensitive reactions in plants, such as tobacco [66]. The molecular mechanism of Rysto-triggered recognition and immunity is poorly understood to date. Thoughtfulness of the indestructible mechanisms in the control of viral recognition is important for understanding the resilience of plants to disease resistance after the translation to effective crop protection. Rysto associates directly with PVY CP in plants that are conditioned by the presence of a CP central 149 amino acids domain. Each deletion that affects the CP core region impairs the ability of Rysto to trigger defense [66].

High-throughput sequencing has brought new possibilities to the diversity and complexity of mixed viral infections. Response to *Potato virus Y* infection differs in different potato cultivars described as tolerance (no indications are visible, but unobtrusive yield loss may be documented), resistance (no indicators develop), or susceptibility (indicators appear). The most cost-effective method to combat PVY infections is the use of resistant plant material.

Virus infection is recognized in plants through RNA silencing in potatoes and other plants as a universal manner for defense against viral attacks, interfering with viral replication in the plant cell. The first protein of PVY found to suppress RNA silencing was HCPro. The P1 protein preceding HCPro in the viral polyprotein may stabilize PVY HCPro and its impact on the suppression of RNA silencing [67]. The relative efficiency of antiviral RNA silencing in a potato plant and the counterdefensive suppression of RNA silencing by the virus determine the amount of virus accumulated in infected tissues.

Introgression of resistant genes from wild potato varieties into commercial cultivars has been successful [68, 69] even though over time there is resistance breakdown noted from the local cultivars, resulting in new PVY infection. Kenyan farmers have been changing potato cultivars over time due to reasons of preference, and the potato farming industry has noted a shift from cultivars Nyayo and Desiree, which were the major cultivars grown in the 1980s and early 1990s [70] to Tigoni, Nyayo, Thimathuti, Dutch Robyjn, Asante, and Shangi varieties being more embraced (MoA/GTZ/PSDA 2009). This study supports the findings in [71] of the main PVY strain prevalence in Kenya to be the recombinant strain type PVY^N:O^. In the future, however, we recommend the use of modern methods that can precisely identify and differentiate PVY strains beyond the four main strains of PVY such as described in [72].

Phylogenetic analysis of sequences from several parts of the world retrieved from the GenBank vis-a-vis our Kenyan whole-genome isolate GenBank accession number PP069009 and a USA counterpart JQ924287 revealed a shared common last ancestor node with isolates from Slovakia (Figure 4). This displayed maximal support at 100% probability. This phylogenetic positioning suggests a close evolutionary relationship between the PVY_Kenya isolate and its counterpart from the United States, potentially tracing back to a common ancestral lineage rooted in Slovakia. Notably, the MT522445.1 sequence of the potato yellow vein virus was included as an outgroup for comparative analysis. Employing the MAFFT alignment tool [42] followed by Bayesian phylogenetic inference through MrBayes [43] within the SATO v 0.1.4 pipeline [73], a phylogenetic tree was constructed (Figure 4). We established a strong provision for a migration trail from South America to Europe (Figure 4). This can be explained by a probable situation, in which PVY was first introduced into Europe from South America, through gene flow from the global trade of potatoes and further possibly spread to Africa.

A mosaic-like genetic structure within the PVY Kenya isolate was evident, with prominent multiple illustrations of gene recombination events. A study done by Gibbs [74] revealed the five distinct phylogroups that all PVY isolates for which complete genome sequences were then known were as a result of recombination from distinct parent phylogroup studies [75, 76]. Recombination is a significant driving force in the evolution and divergence of many plant viruses as supported in the findings of a study in [77] as evident in Figures 3 and 4. This is also demonstrated in Figures 7(a) and 7(b) where acumens of genetic diversity and distribution of PVY strains across different continents (America, Africa, Asia, and Europe) have been illustrated. The diverse array of haplotypes signifies the potential evolutionary divergence or recombination events within the PVY population. The findings of this study are similarly consistent with that of a study [74], which observed that genetic variations of this pathogen have geographically-driven adaptation as a significant factor of the diversity of *Potato virus Y*. Previous studies on PVY in different parts of the world share parental history with where it was first domesticated in the Andean region of South America and spread to other regions through similar historical events [4, 34], which has a shared evolutionary background with Potato virus V [78]. Studies from [9, 79] support most of the viruses (e.g., wild potato mosaic virus, sunflower ring blotch virus, Potato virus V, Peru tomato virus, and Bidens mosaic virus) all of which are from the PVY lineage were isolated from plants native to South America.

Recombinant analysis conducted for the phylogeny of *Potato virus Y* (PVY) revealed a mosaic-like genetic structure within the PVY Kenya isolate. RNA viruses show exciting evolutionary dynamic forces due to large population sizes, short replication cycles, and high mutation rates [80]. The current study hypothesizes that the PVY_Kenya isolate derives genetic material from multiple parental sequences, as evidenced by the reference sequences obtained from the NCBI database. This helps the virus's adaptability to different geographical positions. Phylogenetic positioning and potential historical transmission patterns across continents have been revealed in this study (Figures 7(a) and 7(b)).

Plant pathogens often undergo strong selective pressures that rapidly change depending not only on the vagaries of the ecosystems they inhabit but also on direct inputs from humans [81]. High mutation frequencies determined by mutation and fitness produce quasispecies, which is very common among RNA viral species [82, 83]. The direction and strength of selection can be calculated and categorized as purifying selection, neutral evolution, or positive selection, depending on values obtained from a study. RNA viruses can quickly adapt to changing selective pressures and new hosts. This study revealed pervasive positive/diversifying selection at five specific sites and pervasive negative/purifying selection at 267 sites within the *Potato virus Y* (PVY) genome, each supported by a robust posterior probability (Prob [*α* > *β*] >0.9), unveiling a complex interplay of selective forces shaping the genetic landscape of this viral population. Here, the selection pressures within PVY_Kenya branches suggest a more intricate evolutionary history or potentially different, undetectable types of selective pressures acting on PVY evolution and a relatively low overall genetic diversity within the PVY population, despite its multicontinental origins. Similar to studies done by Gao et al. [68] on the evolutionary history and global spatiotemporal dynamics of *Potato virus Y* in Russia suggest South America was a hub for the domestication of potatoes, which later was spread to the rest of the world (Figures 7(a) and 7(b)). Europe has also played a major role in the spread of many potato viruses. However, due to the rate of recombination scenarios within the PVY, further investigations need to be employed to offer a complete understanding of the evolution of PVY on a global scale.

## 5. Conclusion

*Potato virus Y* impacts negatively on solanaceous crop yield. Despite this, a substantial gap in knowledge is evident, especially regarding the comprehensive genomic status of PVY in Africa, and notably in East Africa. Limited insights into mutations and recombination events within the Kenyan PVY genome compound the outdated understanding of PVY strains affecting potato production in Kenya. Farmers lack vital information on the pathotypes responsible for losses caused by various PVY strains, highlighting the urgent need for further research. This study reveals the dynamic phylogeny of PVY, indicating ongoing recombination activities within the genome that could lead to the emergence of new strains. Molecular tools offer insights into the genomic dynamics of viruses, providing valuable information on PVY's genomic diversity, especially within the economically important virus genus. Our findings, which showcase the first molecular footprint of the Kenyan PVY whole genome (GenBank accession number PP069009), enrich scientific understanding by revealing the molecular phylogeography and evolutionary connections of the PVY_Kenya isolate. Placed within the broader context of global PVY diversity, our study illuminates phylogenetic positioning, historical transmission patterns across continents, and adaptive selection pressures. This underscores its potential contribution to PVY's adaptive evolutionary process. Nevertheless, we emphasize the necessity for further investigations into the precise evolutionary dynamics shaping PVY populations in various geographical regions, particularly in Africa. Such endeavors are crucial for advancing tailored management and control strategies to tackle the challenges posed by PVY on a global scale within the scientific discourse [84, 85].

## Figures and Tables

**Table 1:** RNA concentration of 7 PVY (+ve) samples collected from different potato farm fields in Kenya.

Serial no	Potato sample name	Sample ID	RNA quality	RNA concentration (ng/*µ*l)
1	Kenya Mpya	K4	2.07	481
2	Screen house variety	K12	1.95	72
3	Dutch	K16	1.99	176.5
4	Shangi	K23	2.05	375.9
5	Dutch	K33	1.77	210.9
6	Dutch	K76	1.97	141.3
7	Sherekea	K82	1.79	42

**Table 2:** Primers names and sequence in the sequence in 5′–3′.

Name of primers for CP	Sequence in 5′–3′	Name of primers for P1	Sequence in 5′–3′
YoCP2FOR	AGAGCAAGGCAGCATCCAGT	P1oFOR	CATGGCAACCTACATGTCAACAATC

YoCP2REV	TGCACCGAACCATAAGCCCA	P1REV	AAAATGCATCATTGAGTAACCTTGGAAC
		P1nFOR	CATGGCAACTTACACATCAACAATCC
		P1REV	AAAATGCATCATTGAGTAACCTTGGAAC

**Table 3:** Description of the PVY type found among sampled field accessions from Kiambu, Nyandarua, and Nakuru counties of Kenya.

Sample ID	Natural host	P1-N type	P1 O-N recombinant	Symptoms on leaves	Locality	Year of sampling	PVY type	Accession number
K4	Potato	✓		(i) Mild mosaic (ii) Stunted growth	Tigoni	2018	N or recombinant	OR571473
K33	Potato	✓		(i) Stunted short growth (ii) Yellowing specks on leaves	Gatimu	2018	N or recombinant	OR571477
K12	Potato		✓	(i) Deepening venation (ii) Mild mosaic pattern	Tigoni	2018	Recombinant	OR571474
K23	Potato		✓	(i) Moderate mosaic pattern (ii) Stunted growth	Tigoni	2018	Recombinant	OR571476
K76	Potato		✓	(i) Crinkling of leaves (ii) Rugose mosaic	Marindas subcenter	2018	Recombinant	OR571478
K82	Potato		✓	(i) Mosaic pattern (ii) Deep venation (iii) Small leave sizes	Marindas subcenter	2018	Recombinant	OR571479
K16	Potato	✓	✓	(i) Deep green pigmentation (ii) Deep yellow specs (iii) Small leave size, crinkled	Tigoni	2018	N and recombinant	OR571475

**Table 4:** Haplotype diversity.

Haplotype analysis	
Nucleotide diversity	Pi = 0.00354881
No segregating sites	290
No parsimony-informative sites	53
Tajima's D statistic	*D* = −2.26205

Analysis of molecular variance (AMOVA)	

Genetic differentiation	ø_ST_ = 0.45224 (*p* < 0.001)

Fixation indices	Phi_ST: 0.45224 (*p* < 0.001)
	Phi_SC: 0.39131 (*p*=0.006)
	Phi_CT: 0.10010 (*p*=0.161)

## Data Availability

The accession number of the complete genome sequence of the *Potato virus Y* is PP069009 and is available in the National Centre for Biotechnological Information (NCBI). The accession numbers of partial sequences of the seven *Potato virus Y* strains identified from farmer fields and used in this study are OR571473, OR571477, OR571474, OR571476, OR571478, OR571479, and OR571475 and are available in the National Centre for Biotechnological Information (NCBI). Any other relevant data may be available upon request from the first author.

## References

[1] Kimathi S. M., Ayuya O. I., Mutai B. (2021). Adoption of climate-resilient potato varieties under partial population exposure and its determinants: case of smallholder farmers in Meru County, Kenya. *Cogent Food & Agriculture*.

[2] Scott G. J. A. (2021). Review of root, tuber and banana crops in developing countries: past, present and future. *International Journal of Food Science and Technology*.

[3] Hanˇcinský R., Mihálik D., Mrkvová M., Candresse T., Glasa M. (2020). Plant viruses infecting *solanaceae* family members in the cultivated and wild environments. *A Review. Plants*.

[4] Torrance L., Talianksy M. (2020). Potato virus Y emergence and evolution from the andes of South America to become a major destructive pathogen of potato and other solanaceous crops worldwide. *Viruses*.

[5] Torrance L., Andreev I. A., Gabrenaite Verhovskaya R., Cowan G., Makinen K., Taliansky M. E. (2006). An unusual structure at one end of potato Potyvirus particles. *Journal of Molecular Biology*.

[6] Hofius D., Maier A. T., Dietrich C. (2007). Capsid protein-mediated recruitment of host DnaJ-like proteins is required for *potato virus Y* infection in tobacco plants. *Journal of Virology*.

[7] Sacristan S., Garcia-Arenal F. (2008). The evolution of virulence and pathogenicity in plant pathogen populations. *Molecular Plant Pathology*.

[8] Hull R. (2002). *Matthews’ Plant Virology*.

[9] Gibbs A., Ohshima K. (2010). Potyviruses and the digital revolution. *Annual Review of Phytopathology*.

[10] Rashid M.-O., Wang Y., Han C.-G. (2020). Molecular detection of potato viruses in Bangladesh and their phylogenetic analysis. *Plants*.

[11] Moury B., Desbiez C. (2020). Host range evolution of potyviruses: a global phylogenetic analysis. *Viruses*.

[12] Kimathi S. M., Ayuya O. I., Mutai B. (2021). Adoption of climate-resilient potato varieties under partial population exposure and its determinants: case of smallholder farmers in Meru County, Kenya. *Cogent Food & Agriculture*.

[13] Samarskaya V., Ryabov E., Gryzunov N. (2023). The temporal and geographical Dynamics of potato virus Y diversity in Russia. *International Journal of Molecular Sciences*.

[14] Gibbs A. J., Hajizadeh M., Ohshima K., Jones R. A. C. (2020). The potyviruses: an evolutionary synthesis is emerging. *Viruses*.

[15] Chikh-Ali M., Gray S. M., Karasev A. V. (2013). An improved multiplex IC-RT-PCR assay distinguishes nine strains of Potato virus Y. *Plant Disease*.

[16] Kehoe M. A., Jones R. A. C. (2016). Improving *Potato virus Y* strain nomenclature: lessons from comparing isolates obtained over a 73‐year period. *Plant Pathology*.

[17] Verbeek M., Piron P. G. M., Dullemans A. M., Cuperus C., van der Vlugt R. A. A. (2010). Determination of aphid transmission efficiencies for N, NTN and Wilga strains of Potato virus Y. *Annals of Applied Biology*.

[18] Mondal S., Wenninger E. J., Hutchinson P. J. (2016). Comparison of transmission efficiency of various isolates of potato Virus Y among three aphid vectors. *Entomologia Experimentalis et Applicata*.

[19] Mondal S., Gray S. (2017). Sequential acquisition of potato Virus Y strains by *Myzus persicae* favors the re-emergence of recombinant strains. *Virus Research*.

[20] Kreuze J. F., Souza-Dias J. A. C., Jeevalatha A., Figueira A. R., Valkonen J. P. T., Jones R. A. C., Campos H., Ortiz O. (2020). Viral diseases in potato. *The Potato Crop: Its Agricultural, Nutritional and Social Contribution to Humankind*.

[21] Morante M. C., Salazar E. C., Villegas J. B., Ponce N. T. (2021). Virus incidence associated with native potato yield in microcenters on potato genetic diversity of Bolivia. *American Journal of Potato Research*.

[22] Onditi J., Nyongesa M., van der Vlugt R. (2021). Prevalence, distribution, and control of six major potato viruses in Kenya. *Trop plant Pathol*.

[23] Dupuis B., Nkuriyingoma P., Ballmer T. (2023). Economic impact of potato virus Y (PVY) in Europe. *Potato Research*.

[24] da Silva W., Kutnjak D., Xu Y. (2020). Transmission modes affect the population structure of potato virus Y in potato. *PLoS Pathogens*.

[25] Martinelli F., Scalenghe R., Davino S. (2015). Advanced methods of plant disease detection. A review. *Agronomy for Sustainable Development*.

[26] Shahid M., Sattar M., Iqbal Z., Raza A., Al-Sadi A. (2020). Next-generation sequencing and the CRISPR-cas nexus: a molecular plant virology perspective. *Frontiers in Microbiology*.

[27] Elwan E., Rabie M., Aleem E., Fattouh F. A., Kagda M., Zaghloul H. (2023). Exploring virus presence in field-collected potato leaf samples using RNA sequencing. *Journal of Genetic Engineering and Biotechnology*.

[28] Hu T., Dimitri Monos N. C., Dinh A. (2021). *Next-generation Sequencing Technologies: An Overview*.

[29] Osborne A., Phelan J., Kaneko A. (2023). Drug resistance profiling of asymptomatic and low-density Plasmodium falciparum malaria infections on Ngodhe island, Kenya, using custom dual-indexing next-generation sequencing. *Scientific Reports*.

[30] Novitsky V., Nyandiko W., Vreeman R. (2023). Added value of next generation sequencing in characterizing the evolution of HIV-1 drug resistance in Kenyan youth. *Viruses*.

[31] Luka M., Kamau E., de Laurent Z. (2021). Whole genome sequencing of two human rhinovirus A types (A101 and A15) detected in Kenya, 2016-2018. *Wellcome open research*.

[32] Alfred A., Patrick O., Rose O., Read D., Thompsons G., John M. (2020). Next-generation sequencing platforms for potato virus hunting, surveillance, and discovery. *African Journal of Bacteriology Research*.

[33] Mwatuni A., Nyende A., Njuguna J., Zhonguo X., Machuka E., Stomeo F. (2020). Occurrence, genetic diversity, and recombination of maize lethal necrosis disease-causing viruses in Kenya. *Virus Research*.

[34] Fuentes S., Jones R. A. C., Matsuoka H., Ohshima K., Kreuze J., Gibbs A. J. (2019). Potato virus Y; the Andean connection. *Virus Evolution*.

[35] Martin D., Murrell B., Golden M., Khoosal A., Muhire B. (2015). RDP4; Detection and analysis of recombination patterns in virus genomes. *Virus Evolution*.

[36] Padidam M., Sawyer S., Fauquet C. M. (1999). Possible emergence of new geminiviruses by frequent recombination. *Virology*.

[37] Martin D. P., Posada D., Crandall K. A., Williamson C. (2005). A modified bootscan algorithm for automated identification of recombinant sequences and recombination breakpoints. *AIDS Research and Human Retroviruses*.

[38] Smith J. (1992). Analyzing the mosaic structure of genes. *Journal of Molecular Evolution*.

[39] Posada D., Crandall K. A. (2001). Evaluation of methods for detecting recombination from DNA sequences: computer simulations. *Proceedings of the National Academy of Sciences*.

[40] Gibbs M. J., Armstrong J. S., Gibbs A. J. (2000). Sister-Scanning: a Monte Carlo procedure for assessing signals in recombinant sequences. *Bioinformatics*.

[41] Lam H. M., Ratmann O., Boni M. F. (2018). Improved algorithmic complexity for the 3SEQ recombination detection algorithm. *Molecular Biology and Evolution*.

[42] Katoh K., Misawa K., Kuma K. I., Miyata T. (2002). MAFFT: a novel method for rapid multiple sequence alignment based on fast Fourier transform. *Nucleic Acids Research*.

[43] Huelsenbeck J. P., Ronquist F. (2001). MRBAYES: Bayesian inference of phylogenetic trees. *Bioinformatics*.

[44] Smith M. D., Wertheim J. O., Weaver S., Murrell B., Scheffler K., Kosakovsky Pond S. L. (2015). Less is more: an adaptive branch-site random effects model for efficient detection of episodic diversifying selection. *Molecular Biology and Evolution*.

[45] Wertheim J. O., Murrell B., Smith M. D., Kosakovsky Pond S. L., Scheffler K. (2015). RELAX: detecting relaxed selection in a phylogenetic framework. *Molecular Biology and Evolution*.

[46] Muse S. V., Gaut B. S. (1994). A likelihood approach for comparing synonymous and nonsynonymous nucleotide substitution rates, with application to the chloroplast genome. *Molecular Biology and Evolution*.

[47] Delport W., Scheffler K., Seoighe C. (2008). Models of coding sequence evolution. *Briefings in Bioinformatics*.

[48] Anisimova M., Kosiol C. (2009). Investigating protein-coding sequence evolution with probabilistic codon substitution models. *Molecular Biology and Evolution*.

[49] Kosakovsky Pond S. L., Murrell B., Fourment M., Frost S. D. W., Delport W., Scheffler K. (2011). A random effects branch-site model for detecting episodic diversifying selection. *Molecular Biology and Evolution*.

[50] Murrell B., Wertheim J. O., Moola S., Weighill T., Scheffler K., Kosakovsky Pond S. L. (2012). Detecting individual sites subject to episodic diversifying selection. *PLoS Genetics*.

[51] Murrell B., Moola S., Mabona A. (2013). FUBAR: a fast, unconstrained Bayesian approximation for inferring selection. *Molecular Biology and Evolution*.

[52] Hu X., Karasev A., brown C., Lorenzen J. (2009). Sequence characteristics of potato Virus Y recombinants. *Journal of General Virology*.

[53] Edgar R. C. (2004). MUSCLE: multiple sequence alignment with high accuracy and high throughput. *Nucleic Acids Research*.

[54] Rozas J., Ferrer-Mata A., Sánchez-DelBarrio J. C. (2017). DnaSP 6: DNA sequence polymorphism analysis of large data sets. *Molecular Biology and Evolution*.

[55] Leigh J. W., Bryant D. (2015). POPART: full-feature software for haplotype network construction. *Methods in Ecology and Evolution*.

[56] Manolopoulou I., Legarreta L., Emerson B. C., Brooks S., Tavare S. (2011). A Bayesian approach to phylogeographic clustering. *Interface Focus*.

[57] Excoffier L., Laval G., Schneider S. (2005). Arlequin (version 3.0): an integrated software package for population genetics data analysis. *Evolutionary Bioinformatics*.

[58] Tajima F. (1989). Statistical method for testing the neutral mutation hypothesis by DNA polymorphism. *Genetics*.

[59] Zhan Z., Liu X. F., Gong Y. J., Zhang J., Chung H. S. H., Li Y. (2015). Cloud computing resource scheduling and a survey of its evolutionary approaches. *ACM Computing Surveys*.

[60] Ippc secretariat (2021). *Scientific Review of the Impact of Climate Change on Plant Pests Global challenge to Prevent and Mitigate Plant Risks in Agriculture, Forestry, and Ecosystems*.

[61] Hawkes J. G. (1990). *The Potato: Evolution, Biodiversity, and Genetic Resources*.

[62] Glendinning D. R. (1983). Potato introductions and breeding up to the 20th century. *New Phytologist*.

[63] Nunn N., Qian N. (2010). The Columbian exchange: a history of disease, food, and ideas. *The Journal of Economic Perspectives*.

[64] Funke C. N., Nikolaeva O. V., Green K. J. (2017). Strain-specific resistance to *Potato virus Y* (PVY) in potato and its effect on the relative abundance of PVY strains in commercial potato fields. *Plant Disease*.

[65] Tran L. T., Green K. J., Rodriguez-Rodriguez M. (2022). Prevalence of recombinant strains of potato virus Y in seed potato planted in Idaho and Washington States between 2011 and 2021. *Plant Disease*.

[66] Grech Baran M., witck K., Jaroslaw T. (2022). The Ry_sto_ immune receptor recognizes a broadly conserved feature of potyviral coat proteins. *New pathologists*.

[67] Fernández V., Sotiropoulos T., Brown P. H. (2013). *Foliar Fertilisation: Principles and Practices*.

[68] Gao F., Kawakubo S., Ho S., Ohshima K. (2020). The evolutionary history and global spatio-temporal dynamics of potato virus Y. *Virus Evolution*.

[69] Quenouille J., Vassilakos N., Moury B. (2013). *Potato virus Y*: a major crop pathogen that has provided major insights into the evolution of viral pathogenicity. *Molecular Plant Pathology*.

[70] Crissman C., Crissman M., Carli C. (1993). *Seed Potato Systems in Kenya: A Case Study*.

[71] Moa/Gtz-Psda (2009). *National Potato Taskforce Report 2009. Final Report*.

[72] Onditi J., Nyongesa M., van der Vlugt R. (2022). Prevalence, distribution, and control of potato virus Y (PVY) strains in Kenyan potato cultivars. *Tropical Plant Pathology*.

[73] Wekesa C., Okoth P., Muoma J. (2023). Sato – sequence analysis toolkit. *GitHub*.

[74] Hasiów-Jaroszewska B., Stachecka J., Minicka J., Sowiński M., Borodynko N. (2015). Variability of potato virus Y in tomato crops in Poland and development of a reverse-transcription loop-mediated isothermal amplification method for virus detection. *Phytopathology*.

[75] Gibbs A. J., Ohshima K., Yasaka R., Mohammadi M., Gibbs M. J., Jones R. A. C. (2017). The phylogenetics of the global population of potato virus Y and its necrogenic recombinants. *Virus Evolution*.

[76] Glais L., Bellstedt D. U., Lacomme C. (2017). Diversity, characterization, and classification of PVY. *Potato Virus Y: Biodiversity, Pathogenicity, Epidemiology, and Management*.

[77] Karasev A. V., Gray S. M. (2013). Continuous and emerging challenges of Potato virus Y in potato. *Annual Review of Phytopathology*.

[78] Mao Y., Sun X., Shen J. (2019). Molecular evolutionary analysis of potato virus Y infecting potato based on the VPg gene. *Frontiers in Microbiology*.

[79] Fuentes S., Gibbs A., Adams I. P. (2022). Phylogenetics and evolution of potato virus V: another Potyvirus that originated in the andes. *Plant Disease*.

[80] Fribourg C. E., Gibbs A. J., Adams I. P., Boonham N., Jones R. A. C. (2019). Biological and molecular properties of wild potato mosaic virus isolates from pepino (*Solanum muricatum*). *Plant Disease*.

[81] Glasa M., Hančinský R., Šoltys K. (2021). Molecular characterization of potato virus Y (PVY) using high-throughput sequencing: constraints on full genome reconstructions imposed by mixed infection involving recombinant PVY strains. *Plants*.

[82] Derbyshire M. (2020). Bioinformatic detection of positive selection pressure in plant pathogens: the neutral theory of molecular sequence evolution in action. *Frontiers in Microbiology*.

[83] Sanjuan R. (2008). *Quasispecies. Encyclopedia of Virology*.

[84] Faostat (2023). Crops statistics database of 2022. https://www.fao.org/faostat/en/.

[85] Xu L., Zhang W., Gao Y., Meng F., Nie X., Bai Y. (2022). Potato virus Y strain N-wi offers cross-protection in potato against strain NTN-NW by superior competition. *Plant Disease*.

